# Understanding the Role of Base in Catalytic Transfer Hydrogenation: A Comparative Review

**DOI:** 10.3390/molecules31010064

**Published:** 2025-12-24

**Authors:** Batoul Taleb, Assi Al Mousawi, Ali Ghadban, Ismail Hijazi, Rasha Al Ahmar, Mikhael Bechelany, Akram Hijazi

**Affiliations:** 1 Department of Chemistry and Biochemistry, Faculty of Sciences, Lebanese University, Beirut P.O. Box 6573/14, Lebanon; batoul.taleb96@gmail.com (B.T.); ali.ghadban@liu.edu.lb (A.G.); ismail.hijazi@mu.edu.lb (I.H.); rashaahmar2000@gmail.com (R.A.A.); 2 Chemical Sciences Laboratory (CSL@LIU), Department of Biological and Chemical Sciences, School of Arts and Sciences, Lebanese International University, Beirut P.O. Box 146404, Lebanon; assi.mousawi@liu.edu.lb; 3 Institut Européen des Membranes, IEM, UMR 5635, Université de Montpellier, ENSCM, CNRS, Place Eugène Bataillon, 34095 Montpellier, France

**Keywords:** catalytic transfer hydrogenation, reduction reaction, base-free, additive, hydrogen donor, activity, selectivity

## Abstract

Catalytic transfer hydrogenation (CTH) provides a practical and sustainable approach for reducing unsaturated compounds, serving as an alternative to high-pressure H_2_ in laboratory and fine chemical contexts. This broad reaction class includes asymmetric transfer hydrogenation (ATH), a key strategy in enantioselective synthesis due to its operational simplicity, high stereocontrol, and compatibility with sensitive functional groups. A central variable governing CTH efficiency is the role of bases, which may function as essential activators, co-hydrogen donors, or be entirely absent depending on the catalytic system. This review provides a comparison of base-assisted, base-free, and base-as-co-hydrogen-donor CTH methodologies across diverse metal catalysts and substrates. We highlight how bases such as triethylamine, K_2_CO_3_, and NaOH facilitate catalyst activation, modulate hydride formation, and tune reactivity and selectivity. The dual function of bases in formic-acid-driven systems is examined alongside synergistic effects observed with mixed-base additives. In contrast, base-free CTH platforms demonstrate how tailored ligand frameworks, metal-ligand cooperativity, and engineered surface basicity can eliminate the need for external additives while maintaining high activity. Through mechanistic analysis and cross-system comparison, this review identifies the key structural, electronic, and environmental factors that differentiate base-assisted from base-free pathways. Emerging trends—including greener hydrogen donors, advanced catalyst architectures, and additive-minimized protocols—are discussed to guide future development of sustainable CTH processes.

## 1. Introduction

Hydrogenation is a fundamental transformation in organic chemistry, extensively used in both pharmaceutical and industrial applications. It involves the addition of hydrogen to unsaturated compounds in the presence of a catalyst, converting them into saturated products. Traditionally, in industrial settings, this process predominantly relies on molecular hydrogen under elevated pressure—a method known as direct hydrogenation. However, due to safety concerns and operational limitations associated with the handling of molecular hydrogen—particularly outside large-scale industrial facilities—an alternative strategy known as catalytic transfer hydrogenation (CTH) has gained increasing attention [1]. This method has emerged as a safer and more practical approach. In CTH, hydrogen donors such as secondary alcohols, formic acid, or other hydrogen-rich compounds serve as substitutes for gaseous hydrogen. This strategy eliminates the need for specialized equipment, simplifies reaction setups, and broadens substrate compatibility. The development of catalytic asymmetric transfer hydrogenation (CATH) has further advanced the field, enabling the efficient synthesis of enantiomerically pure compounds through the use of chiral ligands [2,3,4].

The concept of transfer hydrogenation was first introduced in 1925 with the Meerwein–Ponndorf–Verley (MPV) reaction, which involved the reduction of carbonyl compounds using aluminum alkoxides as homogeneous catalysts and alcohols as hydrogen donors [5]. This early method laid the groundwork for future developments but had limitations in terms of efficiency and scope. A significant advancement came with the work of Sasson et al., who introduced the use of late transition metal catalysts in transfer hydrogenation. These catalysts provided much higher yields and broader substrate compatibility compared to the traditional MPV system [6].

While early studies primarily employed homogeneous catalysts, challenges related to separation, recovery, and reuse led to increased interest in heterogeneous systems. A variety of supported metal catalysts have since been developed, demonstrating excellent efficiency in the reduction of various unsaturated compounds such as alkenes, alkynes, and carbonyl groups [7,8].

Over the past decades, CTH has evolved from a laboratory-scale alternative to direct hydrogenation into a central method in academic research and an established strategy in industrial fine chemical and pharmaceutical synthesis. Research has focused on optimizing reaction conditions, developing homogeneous and heterogeneous catalysts [9,10], exploring a wide range of hydrogen donors [11,12], and improving stereoselectivity. As a result, several reviews have examined key aspects of CTH, including catalysts [13], hydrogen donors [14], substrates [15], and mechanistic features. Our previous review [14] focused on hydrogen donors and discussed how donor choice influences catalytic pathways and efficiency. However, despite these advances, the influence of bases—either as additives or absent altogether—has not been comprehensively assessed.

The present review addresses this gap by examining how different bases influence catalytic transfer hydrogenation across both base-assisted and base-free systems. Bases can affect catalyst activation, hydride formation, proton-transfer events, and overall reaction efficiency, yet their contribution is often only briefly noted in the literature. By comparing bases of varying strength and structure, as well as systems that operate without added base, this review emphasizes the practical and mechanistic consequences associated with base choice and reaction environment.

## 2. Role of Base in CTH

The critical role of base in catalytic transfer hydrogenation (CTH) was clearly demonstrated in early work by Chowdhury and Bäckvall in 1991. Using the catalyst [RuCl_2_(PPh_3_)_3_], they showed that the catalytic activity increased by 10^3^–10^4^ upon addition of NaOH [16]. This enhancement was attributed to the base facilitating the dehydrogenation of the hydrogen donor and promoting hydride transfer to the substrate, thereby aiding in its complexation with the metal center.

Notably, amine bases often serve primarily to regulate the acidity and basicity of the reaction medium, as seen in classical systems like the formic acid/triethylamine azeotrope, where triethylamine acts as a buffer. However, in other systems, certain bases may also participate directly in hydrogen transfer, effectively functioning as co-hydrogen donors, and influence the stereoselectivity. A brief summary of the base in CTH is summarized in Table 1.

It should be noted that these functions of the base are not mutually exclusive. In solution, acid–base equilibria allow a given base to simultaneously contribute to hydrogen-donor activation, catalyst activation, proton/hydride transfer, and selectivity control, depending on the reaction conditions.

More recently, significant developments have emerged where certain CTH reactions proceed under base-free conditions. These findings represent a notable advancement, aligning with the principles of green chemistry, which emphasize minimizing the use of auxiliary chemicals to make reactions more sustainable and environmentally friendly.

In this review, the roles of bases in CTH are discussed under three categories: as additives that modulate the reaction environment, as co-hydrogen donors contributing to hydride transfer, and under base-free conditions where catalytic activity occurs without added bases.

### 2.1. Base as Additives

Bases play a fundamental and often indispensable role in catalytic transfer hydrogenation (CTH) reactions. Numerous studies have demonstrated that many CTH systems exhibit a strict dependence on base additives; in several cases, the transformation does not proceed at all without a base, clearly establishing its essential mechanistic function. Furthermore, many catalytic systems display pronounced base sensitivity, whereby efficient hydrogenation is achieved only with specific bases, while others suppress or fail to promote activity—an effect widely reported in the literature. This promoting influence has been particularly well documented in Ir- and Rh-catalyzed CTH reactions [22,23], and similar trends have been observed across a wide range of ruthenium-based catalytic systems [24].

**Scheme 1:**
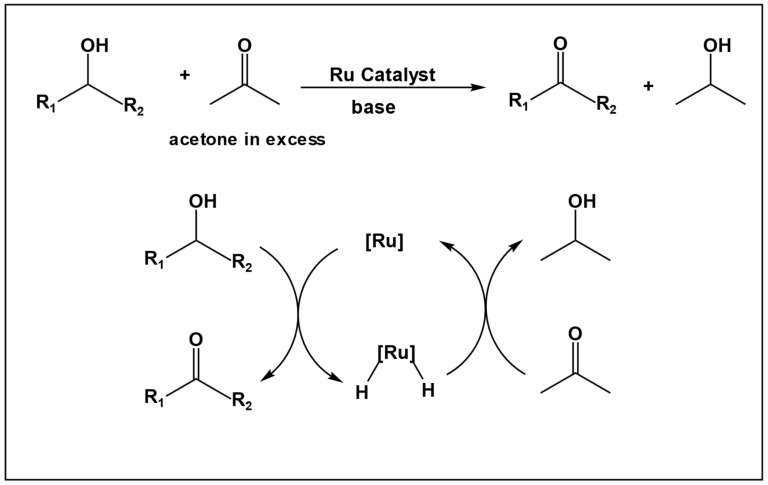
Catalytic Oppenauer-type oxidation.

The importance of the base becomes even more evident in CTH systems involving secondary alcohols as hydrogen donors in the presence of a transition-metal catalyst. The redox relationship between the alcohol and the ketone is inherently reversible, meaning that the reaction can shift toward either reduction or oxidation depending on relative concentrations. When the ketone is in excess, it tends to function as a hydrogen acceptor, favoring the competing Oppenauer-type oxidation pathway (Scheme 1) [25]. In these reversible systems, the base serves several essential functions: it activates the alcohol via deprotonation, promotes hydride transfer, and shifts the equilibrium toward the desired hydrogenation pathway.

This mechanistic dependence is well illustrated by the work of G.-Z. Wang et al., who reported that RuCl_2_(PPh_3_)_3_ in acetone achieved complete conversion within 1 h at 56 °C only when 0.1 mmol K_2_CO_3_ was present. Without a base, however, the reaction completely stagnated (<1% conversion even after 6 h) [26]. Such findings confirm that the base is not simply beneficial but required to drive hydrogen transfer and sustain catalytic activity in reversible CTH systems.

**Scheme 2:**
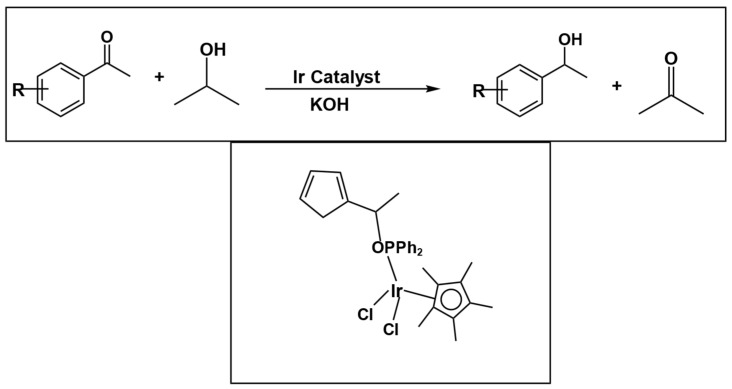
Catalytic Transfer Hydrogenation of Acetophenone Using Iridium Catalyst Using KOH as a Base Additive.

A similar dependence on base activation was observed by Duygu Elma Karakaş et al. [27], who investigated the transfer hydrogenation of acetophenone using isopropanol as the hydrogen donor. Acetophenone is widely used as a model substrate in catalytic transfer hydrogenation because its aromatic ketone structure allows clear evaluation of catalyst activity, and it has industrial relevance in fragrances, pharmaceuticals, and fine chemicals. Their results showed that significant conversion was achieved only when both the catalyst and KOH were present (Scheme 2), further demonstrating that base additives are essential for enabling efficient alcohol-mediated hydrogen transfer.

**Scheme 3:**
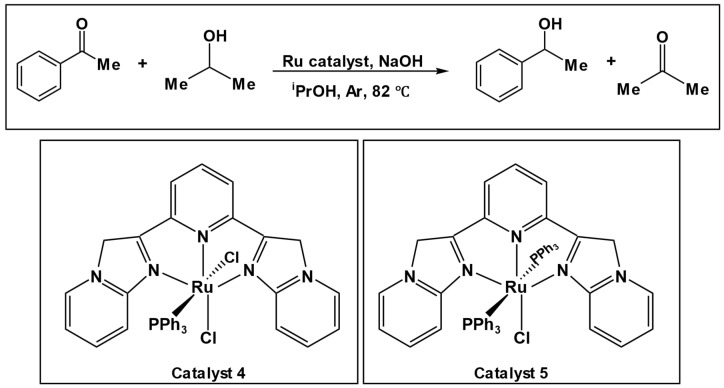
Transfer Hydrogenation of Acetophenone Using NaOH as the Optimal Base.

The results reported by Ke Li et al. [28] highlight the crucial role of the base in enabling catalytic transfer hydrogenation of acetophenone. In the absence of base, no reaction was observed, confirming that base-assisted catalyst activation is essential for reaction progress. Comparison of catalysts 4 and 5 (Scheme 3) further demonstrates the sensitivity of the system to the ligand environment. Catalyst 4 contains two labile chloride ligands that can be readily abstracted by the base, facilitating rapid formation of the active alkoxide species. In contrast, catalyst 5 possesses only one chloride ligand, with the second coordination site occupied by the more sterically hindered PPh_3_ ligand, which may slow catalyst activation and hydrogen transfer. In addition, differences in solubility between the two catalysts may also contribute to their distinct catalytic performances.

The influence of different bases on reaction efficiency was evaluated at a short reaction time of 1 min using catalyst 4 (Table 2). Yields varied from 68% to 96%, reflecting the combined effects of steric hindrance, cation identity, and base strength. Bulky tBuOK gave the lowest yield (68%) due to slower metal–alkoxide formation, while sodium alkoxide (tBuONa, 76%) slightly outperformed its potassium analogue because of stronger Na^+^–alkoxide ion pairing. Strong, unhindered inorganic hydroxides (KOH, NaOH) enabled the fastest activation and highest yields (96%). These results illustrate that, at early reaction times, the base structure and identity critically determine the rate of metal–alkoxide formation and hydrogen-transfer efficiency.

**Scheme 4:**
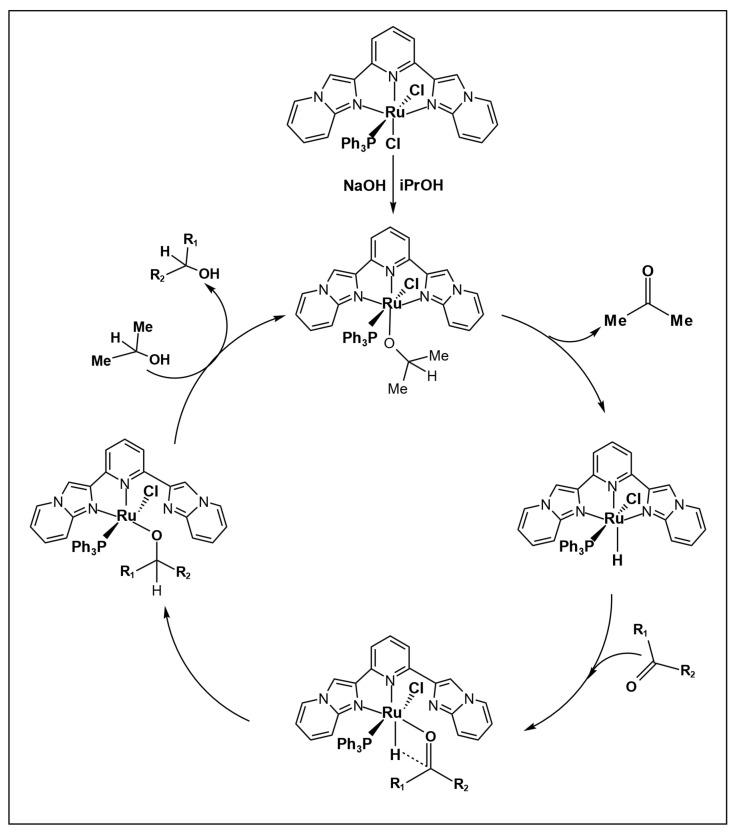
Proposed Base-Assisted Mechanism for Transfer Hydrogenation of Ketones.

The author of Several studies have proposed a mechanism for the transfer hydrogenation of ketones catalyzed by transition metal complexes, in which the base plays a crucial role [29,30,31,32,33]. The reaction begins with the metal complex reacting with the base and a secondary alcohol (e.g., isopropanol) to form a metal–alkoxide intermediate, facilitated by base-mediated deprotonation and coordination to the metal. The alkoxide undergoes β-hydride elimination, generating the active metal–hydride species and releasing acetone, which then transfers hydrogen to the ketone. The ketone coordinates to the metal, allowing hydride transfer from the metal–hydride to the carbonyl group, forming a metal-bound alkoxide intermediate. Finally, the base enables an alcohol exchange step, regenerating the initial alkoxide and completing the catalytic cycle (Scheme 4) [28]. Without the base, formation of the metal–alkoxide and regeneration steps are hindered, resulting in minimal or no catalytic activity.

**Scheme 5:**
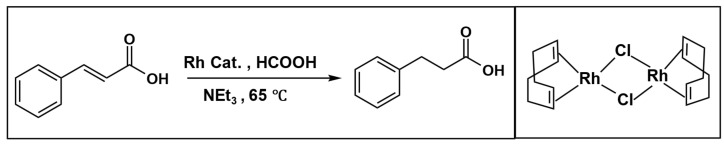
Rhodium-Catalyzed Hydrogenation of Cinnamic Acid Using Triethylamine as a Base Additive.

In addition to the commonly studied inorganic bases, organic amines have proven to be key additives in facilitating hydrogenation reactions of unsaturated compounds, including alkenes and carbonyls. In our previous work on the rhodium-catalyzed hydrogenation of cinnamic acid, triethylamine was essential for high reactivity, providing a 95% yield, whereas KOH afforded only 4%. This difference can be attributed to the nature of the bases: KOH, a strong Brønsted base, is poorly soluble and may disrupt the coordination sphere of the rhodium dimer or promote catalyst deactivation. In contrast, triethylamine, a weaker Brønsted base and moderate Lewis base, is sterically compatible, fully soluble, and maintains a homogeneous environment that supports catalyst activation and turnover (Scheme 5) [34].

**Scheme 6:**
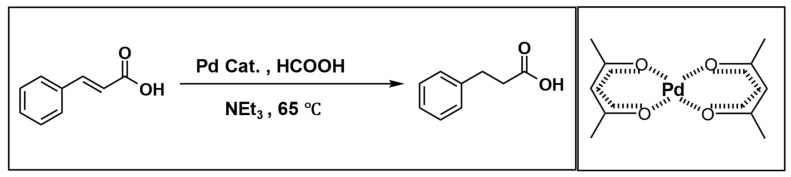
Palladium-Catalyzed Hydrogenation of Cinnamic Acid Using Triethylamine as a Base Additive.

In the Pd(acac)_2_ system [35] (Scheme 6), the catalytic performance correlates strongly with the basicity of the additive. Triethylamine provided a high yield (95%), consistent with its moderate basic strength, which is sufficient to promote formate deprotonation and generate the active Pd–H species required for cinnamic acid reduction. Additionally, triethylamine can partially deprotonate cinnamic acid, increasing its reactivity toward hydride transfer. KOH, being a much stronger base, also delivered a high yield (87%), in line with its ability to accelerate hydride formation through rapid formate activation. In contrast, Na_2_CO_3_ gave only 63% yield, reflecting its substantially weaker basicity and consequently slower formation of reactive hydride intermediates. These results demonstrate that stronger or more readily deprotonating bases enhance Pd-catalyzed transfer hydrogenation, whereas weaker bases limit hydride formation and reduce overall efficiency.

**Scheme 7:**
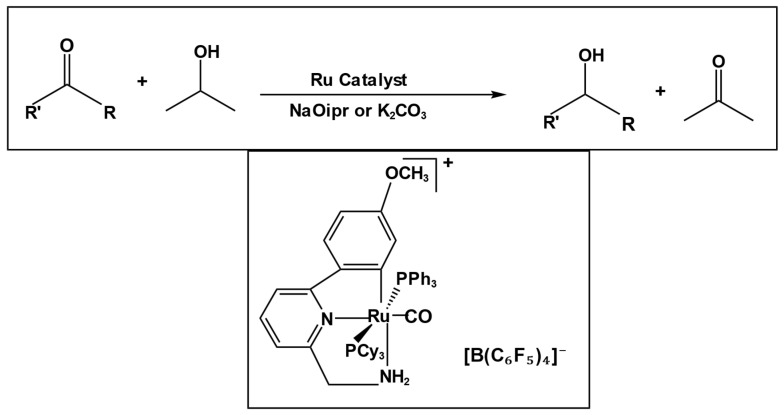
Transfer Hydrogenation of Furfural to Furfuryl Alcohol Using NaOiPr or K_2_CO_3_ as a base additive.

**Scheme 8:**
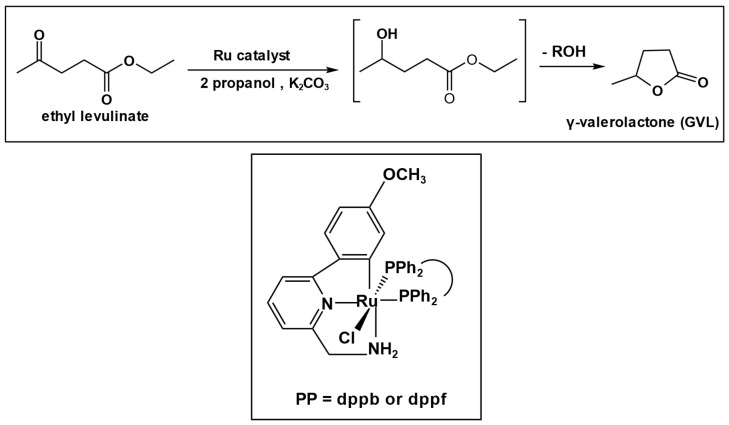
Transfer Hydrogenation of Ethyl Levulinate to γ-Valerolactone using K_2_CO_3_ as a base additive.

Building on the influence of bases discussed earlier, the effect of base strength on both reaction rate and selectivity becomes evident in several Ru-catalyzed systems. Strong bases such as sodium isopropoxide (NaOiPr) generally promote higher conversion rates in catalytic transfer hydrogenation (CTH). For example, the reduction of furfural to furfuryl alcohol using Ru complexes achieved 94% conversion within 30 min when 2 mol% NaOiPr was employed as the base. In contrast, the use of the weaker base potassium carbonate (K_2_CO_3_, 5 mol%) under similar conditions led to only 47% conversion after 24 h, indicating slower reaction kinetics with weaker bases (Scheme 7). However, weaker bases can also offer advantages in certain reactions. In the transfer hydrogenation of ethyl levulinate to γ-valerolactone (GVL) catalyzed by Ru catalyst, K_2_CO_3_ (5 mol%) yielded a slightly higher conversion of 97% in just 20 min compared to 92% conversion in 60 min with NaOiPr. Additionally, K_2_CO_3_ enabled the selective reduction of cinnamaldehyde to allylic alcohol with 94% conversion over 8 h, demonstrating improved selectivity over prolonged reaction times (Scheme 8) [36]. These variations in activity and selectivity suggest that the base may play a more intricate role in the catalytic cycle than merely serving as a reaction medium component.

Beyond simply accelerating reactions, bases in CTH systems can function in a similar manner to a co-catalyst. In alcohol-mediated transfer hydrogenation, the base facilitates reversible hydrogen transfer equilibria between the donor alcohol and the carbonyl substrate—for example, between isopropanol and the ketone being reduced, producing acetone as the oxidized counterpart. By deprotonating the alcohol to form a reactive alkoxide species, the base not only enables efficient hydrogen transfer but also helps regenerate the hydrogen donor continuously. This participation allows the base to influence both the direction and efficiency of the reaction, acting beyond a mere additive and shaping the overall catalytic cycle.

The development of catalytic transfer hydrogenation systems based on inexpensive, earth-abundant metals such as iron, cobalt, nickel, and manganese has become increasingly important as the field seeks alternatives to the precious-metal catalysts traditionally used in reduction chemistry [37]. From an industrial standpoint, these non-noble metals offer clear advantages: lower cost, broad availability, and improved sustainability, all of which are critical considerations for large-scale hydrogenation processes in the fine-chemical and pharmaceutical sectors.

A study by Samanta, Yadav et al. [38] on the cobalt-catalyzed transfer hydrogenation of acetophenone showed a clear dependence of catalytic performance on the strength and steric profile of the base (Table 3). Strong inorganic bases such as KOH (pK_a_ ≈ 14) and NaOH (pK_a_ ≈ 15) provide the highest conversions, with KOH achieving >99% yield, consistent with its ability to promote efficient formation of the active cobalt–hydride species in isopropanol. KOtBu, although even stronger (pK_a_ ≈ 18), affords only moderate yield, suggesting that excessive basicity or steric bulk can disrupt productive metal–ligand activation or facilitate catalyst deactivation. In contrast, the weaker and more sterically encumbered tertiary amine NEt_3_ (pK_a_ ≈ 10.75) gives very low conversion, indicating that insufficient deprotonation of the alcohol limits hydride generation. Overall, these results demonstrate that optimal activity is achieved with moderately strong, non-bulky hydroxide bases, whereas overly strong, highly hindered, or weak bases significantly diminish catalytic efficiency.

A representative example of non-noble metal catalysis is provided by the work of Zheng Wang et al., who examined a manganese pincer complex for the transfer hydrogenation of carbonyl compounds and systematically evaluated a broad range of bases [39]. The reaction showed a pronounced dependence on base identity, reflecting the requirement for efficient generation of the Mn–alkoxide/Mn–hydride species that drive the β-hydride transfer step. Among the bases tested, the sodium alkoxides consistently delivered the highest conversions, with t-BuONa giving 99%. This trend is attributable to the tighter ion pairing of Na^+^, which enhances the deprotonation step needed to form the catalytically active Mn–alkoxide intermediate. Potassium analogues provided slightly lower conversions, consistent with the weaker ion pairing and reduced activation efficiency associated with the larger K^+^ cation. Non-nucleophilic bases such as NaHMDS also performed well, whereas Ca^2+^ and Ba^2+^ hydroxides were ineffective due to limited solubility and insufficient basicity under the reaction conditions. Overall, the results (Table 4) indicate that strong, accessible bases paired with smaller alkali-metal cations are best suited for promoting high activity in Mn-catalyzed transfer hydrogenation.

To address some of the limitations associated with homogeneous CTH systems, several studies have also explored the use of heterogeneous catalysts. These systems were often developed to overcome challenges such as catalyst deactivation, poor recyclability, yet they similarly relied on base additives to achieve efficient hydrogen transfer—further underscoring the importance of this aspect in CTH chemistry. To highlight this expanded perspective, the table below (Table 5) compiles a selection of representative homogeneous and heterogeneous CTH systems, the bases employed, and the corresponding mechanistic implications.

### 2.2. Base as a Co-Hydrogen Donor

In certain transfer-hydrogenation systems, the base does more than simply activate traditional hydrogen donors such as isopropanol—it can also participate directly in the hydrogen-transfer step by generating a reactive hydride-donating species. For example, when a base reacts with formic acid or amine–boranes, it converts them into formate salts (HCOO^−^M^+^) or amidoboranes, which subsequently decompose to release hydride equivalents (HCOO^−^ → CO_2_ + H^−^). In this way, the base indirectly becomes a co-hydrogen donor through in situ formation of the true hydrogen-releasing anion. This concept has been demonstrated in previous studies [48], where base-derived species mediate hydride delivery within the catalytic cycle.

A recurring challenge in asymmetric transfer hydrogenation (ATH) is the decrease in enantiomeric purity observed when isopropanol is used as the hydrogen donor [49,50]. To address this limitation, formic acid in combination with a weak base such as triethylamine has been widely adopted. While this system enables efficient hydrogen transfer under mild and often aqueous conditions, its performance is highly sensitive to the formic acid–to–triethylamine (F/T) ratio, which directly governs hydride generation, reaction kinetics, and stereochemical outcomes.

This behavior has been independently demonstrated in Ru–TsDPEN–catalyzed ketone reduction by Xiaowei Zhou et al. [51] and in aqueous Rh–(1S,2S)–TsDPEN–catalyzed imine reduction studied by Vaishali S. Shende et al. [52]. In both studies, excessively high F/T ratios (acidic conditions) suppress hydride formation due to extensive protonation of the amine, leading to pronounced induction periods and slow initial rates. Conversely, very low F/T ratios (basic conditions) limit the availability of formic acid as the hydrogen source, resulting in reduced overall conversion despite efficient base-mediated activation. Thus, the observed induction periods, rate maxima, and conversion profiles in both systems can be attributed to the same fundamental acid–base balance governing active metal–hydride generation.

**Figure 1:**
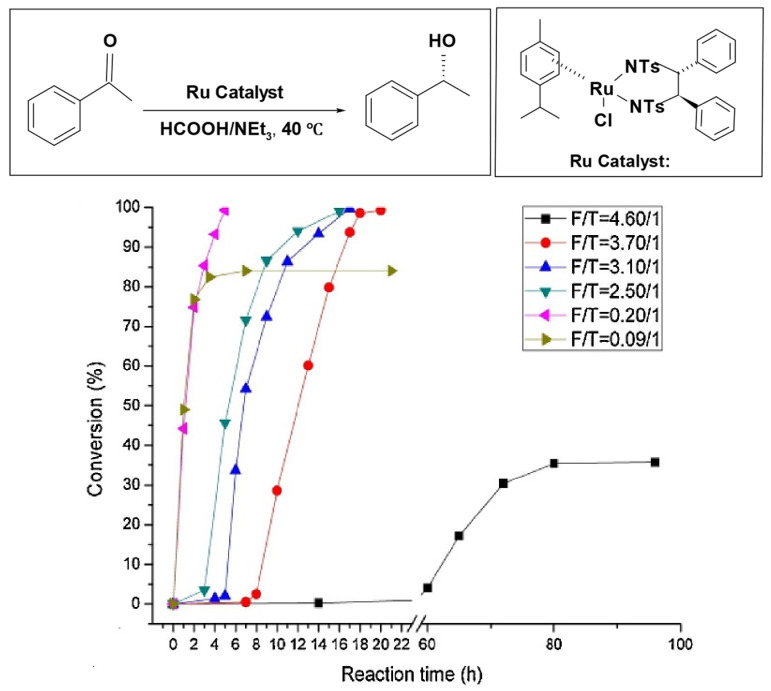
Effect of F/T Ratio on ATH Conversion of Acetophenone.

**Figure 2:**
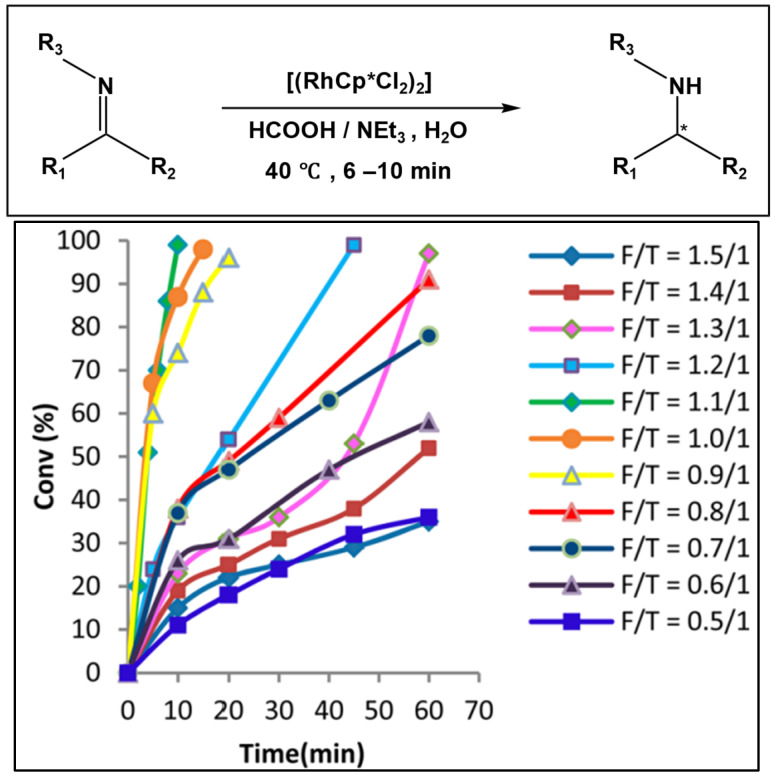
Asymmetric transfer hydrogenation of imines in water in F-T mixtures at different initial F/T ratios. The asterisk (*) denotes the asymmetric (chiral) carbon in the ligand and in the product.

For the Ru–TsDPEN system specifically, Zhou et al. showed that intermediate F/T ratios (e.g., 0.20–0.25/1) enable rapid Ru–H formation, eliminate the induction period, and deliver full conversion within a few hours with consistently high enantioselectivity (ee ≈ 97%) (Figure 1) [51]. At higher F/T ratios, slow accumulation of the active hydride species results in extended induction periods and diminished early-stage ee values, while at very low ratios the reaction becomes hydrogen-donor limited. Shende et al. reported analogous behavior for Rh-catalyzed imine reduction, where maximum activity occurs near F/T ≈ 1.1, and deviations toward either more acidic or more basic conditions again reduce reaction efficiency (Figure 2) [52]. These parallel trends underscore that sensitivity to the F/T ratio is a general characteristic of ATH systems employing formic acid–amine mixtures.

**Scheme 9:**
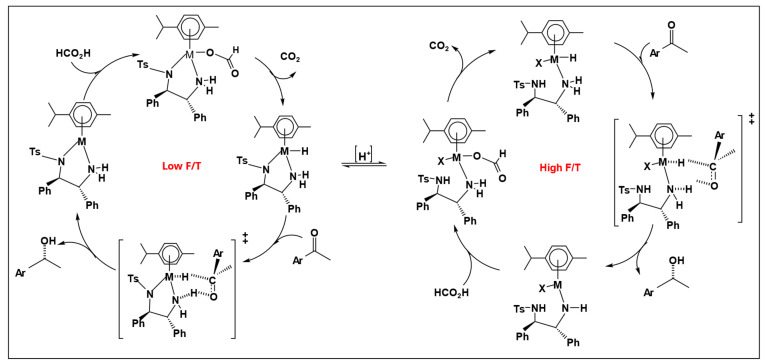
Mechanistic pathways in neutral and acidic F/T media. Adapted from Zhou et al. [51].

The mechanistic origin of these effects is summarized in (Scheme 9). Under basic to near-neutral conditions (low to intermediate F/T ratios), triethylamine efficiently deprotonates formic acid, promoting the formation of a metal–formate intermediate, which subsequently undergoes β-hydride elimination to generate the catalytically active Ru–H complex. The absence of an induction period and the consistently high ee values observed under these conditions indicate that hydrogen transfer proceeds through a well-defined six-membered transition state responsible for stereocontrol. In contrast, under acidic conditions (high F/T ratios), excess formic acid suppresses base-assisted activation, consequently slowing the formation of the Ru–H intermediate and leading to extended induction periods and reduced early-stage enantioselectivity. Thus, the balance between acid and base directly determines the population of active catalytic species and, consequently, the hydrogen transfer rate. Overall, this mechanistic pathway highlights how subtle variations in the F/T ratio can modulate both the kinetics and stereochemical outcome of ATH reactions.

Importantly, while the optimal F/T ratio differs between ketone and imine substrates, the underlying mechanistic framework remains the same, emphasizing the central role of acid–base balance in controlling ATH reactivity and selectivity.

Beyond the F/T ratio, the identity and structure of the base itself significantly influence reaction outcomes in aqueous ATH. Marek Kuzma et al. investigated the effect of different amine bases on the asymmetric transfer hydrogenation of (R)-1,4-dimethyl-3,4-dihydroisoquinoline using formic acid as the hydrogen source in water [21]. Both the basicity (pKa) and molecular structure were found to be key determinants of diastereoselectivity (Table 6). Among tertiary amines, DABCO (pKa 8.7) gave the highest diastereoselectivity (63.8%), outperforming the more basic triethylamine (10.8, 55.5%) and DIPEA (11.4, 46.1%). The lower selectivity of DIPEA was attributed to steric hindrance from its bulky isopropyl groups, which limit interaction with the catalyst, whereas DABCO’s rigid structure allowed more effective coordination with the metal center.

For secondary amines, the trend pyrrolidine (11.2, 71.5%) > morpholine (8.3, 65.5%) ≈ piperidine (11.0, 65.1%) was observed. The higher selectivity of pyrrolidine can be explained by its compact ring and high basicity, supporting efficient hydrogen transfer. Morpholine, despite being less basic, still gave good results, possibly due to stabilization from its oxygen atom through weak hydrogen bonding.

In the case of aromatic bases, pyridine (5.2, 69.3%) showed unexpectedly high selectivity, probably due to coordination of its lone pair to the metal center, while pyrrole (≈0, 65.5%) performed well despite its very low basicity, possibly through π or N–H interactions. Imidazole (7.0) was inactive, which may result from strong coordination that blocks the active site.

Together with Shende’s observations on the sensitivity of aqueous ATH to F/T ratios, Kuzma’s results highlight that both the acid–base balance and the nature of the base govern active metal–hydride formation, reaction kinetics, and stereochemical outcomes. Optimal conditions therefore require not only the correct F/T ratio but also a base capable of effective deprotonation, appropriate coordination, and minimal steric hindrance, particularly in aqueous media. This unified perspective emphasizes the interplay between pH, base identity, solvation, and substrate–catalyst interactions in controlling the efficiency and selectivity of ATH reactions in water.

Thus, in aqueous ATH systems, both the F/T ratio and the choice of base must be optimized together to achieve maximal activity and selectivity.

**Scheme 10:**
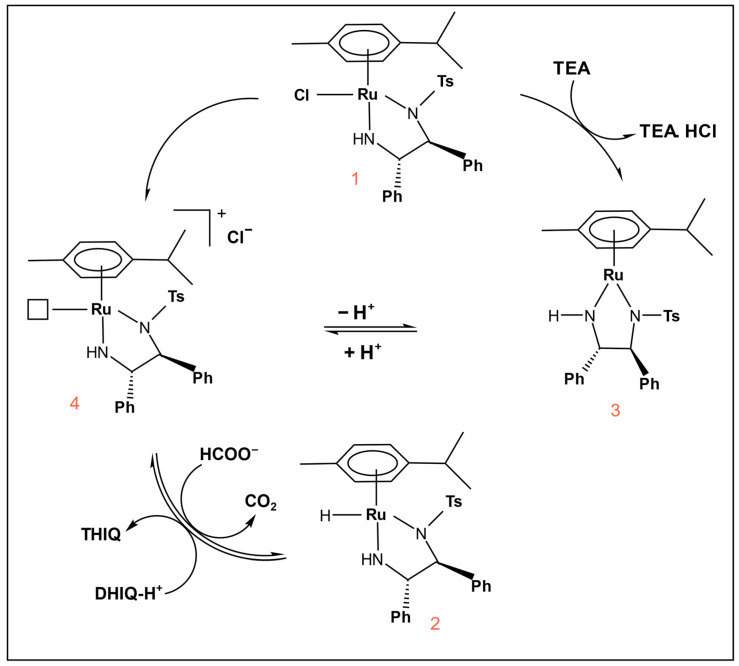
Base-Assisted Mechanism for Asymmetric Transfer Hydrogenation with Ru–TsDPEN Catalyst.

A mechanism commonly reported in the literature describes how asymmetric transfer hydrogenation (ATH) with transition metal complexes proceeds through base-assisted catalyst activation and hydride formation [53,54,55,56] (Scheme 10). The asymmetric transfer hydrogenation (ATH) using the ruthenium catalyst [RuCl(η^6^-p-cymene)TsDPEN] requires a base such as potassium tert-butoxide (t-BuOK) or triethylamine (TEA) to activate the catalyst. The base removes the chloride ligand from the catalyst, forming a reactive ruthenium complex. In the presence of formic acid, this complex reacts to generate a ruthenium hydride species, which serves as the active intermediate in the reaction. This ruthenium hydride transfers hydrogen to prochiral ketones or imines, reducing them to their corresponding chiral alcohols or amines. After the transfer, the catalyst is regenerated by reacting with formate ions from formic acid, completing the catalytic cycle. This mechanism allows efficient and selective hydrogenation under mild conditions.

Marcelo Vilches-Herrera et al. investigated the transfer hydrogenation of benzonitrile using 5 mol% Pd/C with various HCOOH/NEt_3_ ratios [57]. As different acid–base combinations are known to significantly influence activity and selectivity in the transfer hydrogenation of ketones [58], several molar ratios were examined. Even at a high HCOOH/NEt_3_ ratio of 37:1 (corresponding to approximately 20 equivalents of base per mole of catalyst), full conversion and a 98% yield of benzylamine were obtained. In contrast, no reaction occurred without a base, confirming that base-assisted activation of the HCOOH/amine donor system is essential. At a fixed ratio of 18.5:1, the authors compared different amine bases and found that all promoted the reaction to varying degrees (Table 7).

The observed trend reflected differences in steric hindrance and coordinating ability among the bases. Triethylamine and N-butylamine afforded the highest yields (both 98%), consistent with their moderate basicity and limited steric demand, which allow efficient activation of the formic acid donor without interfering with the Pd surface. In comparison, bulkier or more strongly coordinating bases such as DBU (74%), N-methylpyrrolidine (79%), and Hunig’s base (77%) showed reduced activity, likely due to partial catalyst inhibition or slower hydride-transfer steps. Overall, the data indicate that the best performance arises from moderately basic, non-binding amines that balance donor activation with minimal disruption of catalytic turnover.

In a comprehensive review, Gamez et al. detailed the design, synthesis, and catalytic applications of tethered Ru(II)/N-tosyl-1,2-diphenylethylene-1,2-diamine (TsDPEN) complexes, widely employed in asymmetric transfer hydrogenation and the hydrogenation of ketones and imines [59]. The authors examined mechanistic aspects in depth and presented illustrative examples, including a comparative table summarizing the reduction of various acetophenone derivatives using tethered catalysts with HCOOH/NEt_3_ as the hydrogen source.

**Figure 3:**
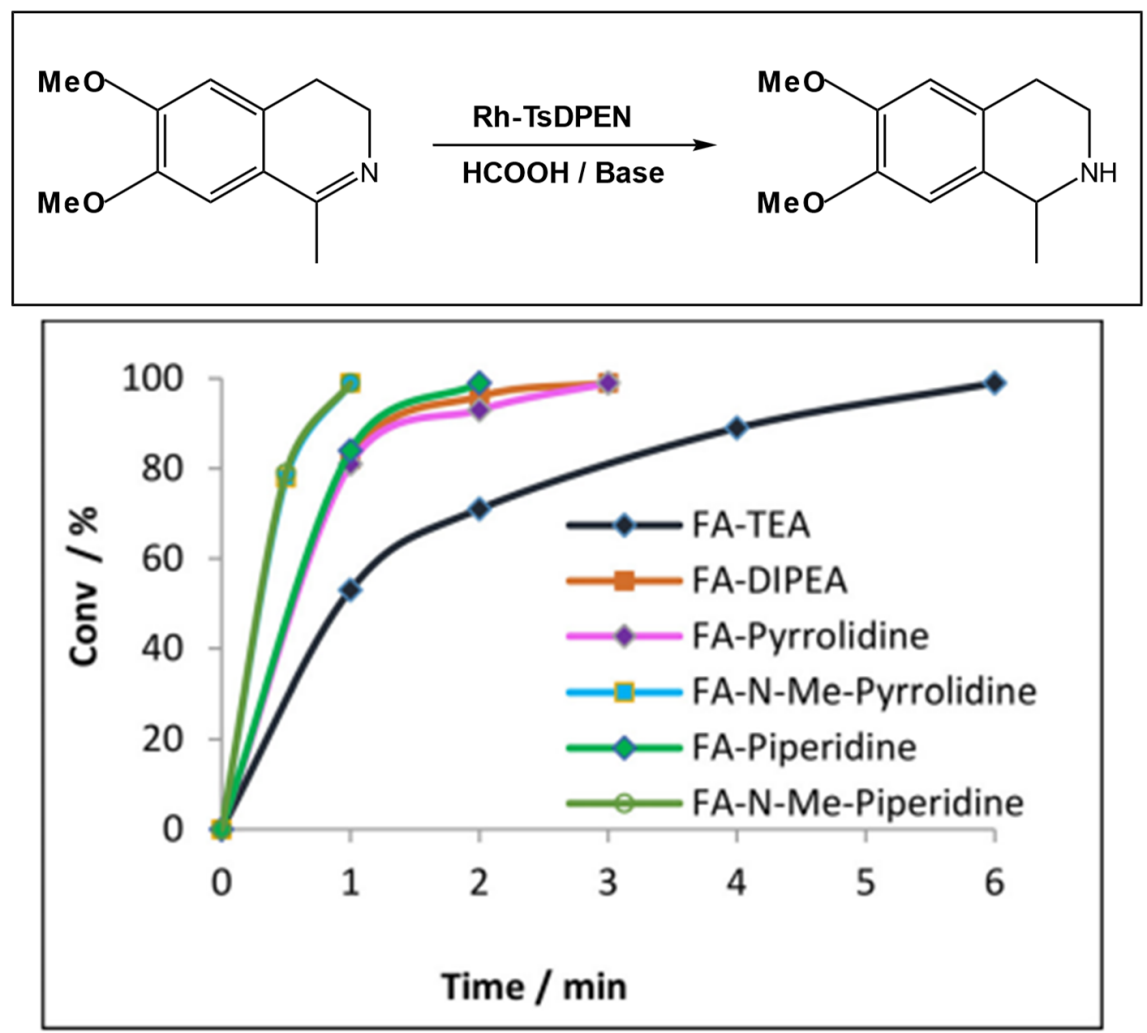
Effect of Base Type on the Conversion in ATH of Imines.

A recent study done by Vaishali S. Shende investigated the asymmetric transfer hydrogenation (ATH) of imines using various bases combined with formic acid as the hydrogen donor at a 1:1 ratio [60] (Figure 3). The conversion over time for several bases is shown in the figure below. Tertiary amines, particularly cyclic ones such as N-methylpyrrolidine and N-methylpiperidine, demonstrated the highest activity and enantioselectivity, achieving near-complete conversion with up to 91% ee within minutes, compatible with previous studies [61,62]. Secondary amines exhibited moderate performance, whereas aromatic bases like imidazole showed low activity, likely due to their lower basicity.

Steric and electronic properties of the bases were identified as crucial factors influencing both reaction rate and selectivity. Computational studies further suggested that bases interact with the catalytic complex via hydrogen bonding, competing with the substrate and thereby affecting both the reaction rate and enantioselectivity [61,62].

Similarly, numerous studies have shown that both the identity of the base and the acid–base ratio strongly influence transfer hydrogenation outcomes, affecting not only conversion and reaction rate but often enantioselectivity as well [63,64,65,66,67]. Across diverse substrate classes, it has become clear that tuning the basic additive—whether it directly participates in hydride delivery, as in formate-based systems, or simply modulates the catalytic environment—remains a decisive factor for achieving optimal activity and selectivity.

**Scheme 11:**
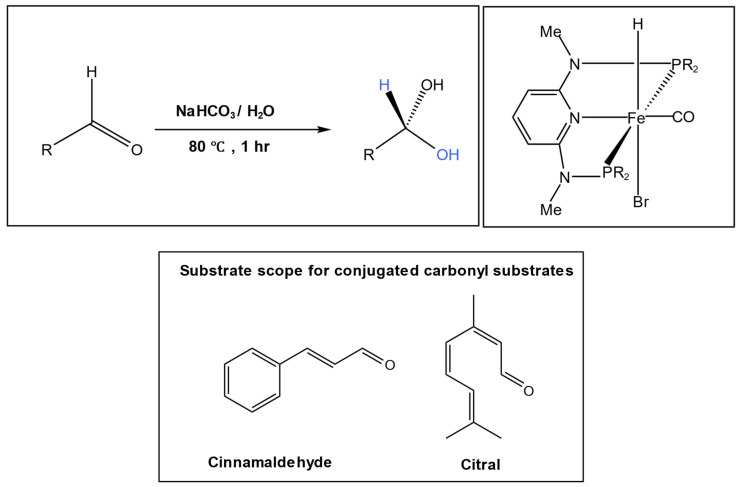
The reduction of an aldehyde in the presence of sodium formate as a hydrogen donor.

The work of Gorgas et al. [48] provides a representative illustration of this concept. Using sodium formate in aqueous medium, they achieved efficient and chemoselective reduction of aldehydes to alcohols under mild conditions (Scheme 11), while sensitive functionalities such as C=C double bonds remained unreacted. This outcome reflects the intrinsic behavior of formate as a controlled, metal-assisted hydride donor: it transfers hydride selectively to the carbonyl group without promoting undesired reductions. These findings strongly support the broader mechanistic principle that when the base generates a stable, well-behaved hydride-releasing anion, it effectively functions as a co-hydrogen donor within the catalytic cycle.

### 2.3. Base-Free Catalytic System

After discussing the essential roles of bases both as additives and co-hydrogen donors in promoting catalytic transfer hydrogenation, it is important to highlight that this requirement can pose challenges, especially when working with base-sensitive substrates or aiming for simpler reaction conditions. Base-free CTH systems eliminate the need for such additives, offering a more straightforward and potentially greener approach. Despite their clear advantages, catalysts capable of efficient transfer hydrogenation under base-free conditions remain relatively limited. Exploring these systems is therefore critical for expanding the versatility and practical applicability of transfer hydrogenation methodologies.

A study by N. Visagie et al. [68] reported a base-free catalytic transfer hydrogenation (CTH) of acetophenone using a Ru catalyst, achieving ~90% conversion within 3 h. The method was successfully applied to a wide range of substrates, giving good to excellent conversions.

**Scheme 12:**
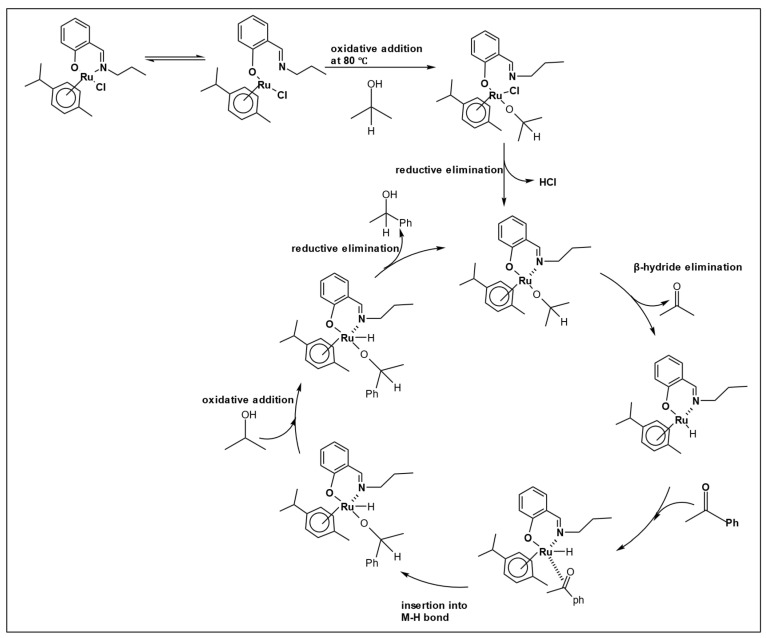
Proposed base-free transfer hydrogenation mechanism with a Ru catalyst.

A base-free transfer hydrogenation mechanism was proposed involving an inner-sphere oxidative addition pathway (Scheme 12) [68]. Initially, the ruthenium catalyst reacts with isopropanol through oxidative addition, forming a ruthenium(IV) isopropoxide intermediate. This step involves breaking the ruthenium–nitrogen bond, as the ruthenium–oxygen bond is stronger and more stable. It is worth noting that, although the Ru–N bond undergoes reversible coordination during the catalytic cycle, the nitrogen donor does not participate in proton transfer. Although the Ru–N bond is hemilabile and coordinates reversibly during the catalytic cycle, the nitrogen donor acts solely as a Lewis base by coordinating to the metal center and does not participate in Brønsted base activity such as proton transfer. Thus, while the ligand facilitates coordination dynamics, it does not contribute to proton transfer steps in the catalytic mechanism. All hydride and proton transfers occur at the ruthenium center, confirming that this pathway is truly base-free. The intermediate then undergoes reductive elimination, releasing HCl and forming a ruthenium(II) species. Next, β-hydride elimination occurs, generating a ruthenium-hydride complex and acetone as a by-product. The substrate, such as acetophenone, coordinates to the metal center by cleaving the ruthenium–nitrogen bond again, leading to the insertion of the ketone into the Ru–H bond and forming a ruthenium-alkoxide intermediate. Another isopropanol molecule then coordinates via oxidative addition, producing a second ruthenium(IV) intermediate. Finally, reductive elimination regenerates the ruthenium (II) catalyst and releases the hydrogenated product. This mechanism allows the transfer hydrogenation to proceed efficiently without the need for an external base.

**Scheme 13:**
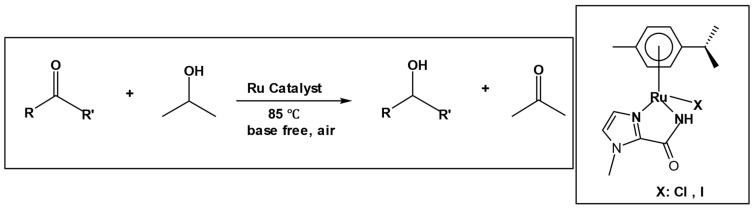
Catalytic transfer hydrogenation of ketones using a ruthenium catalyst under base-free conditions.

A major limitation of most reported transfer hydrogenation catalysts is their dependence on an inert atmosphere and/or the presence of an external base to generate the active metal–hydride species. Such requirements not only complicate the reaction setup and workup but also restrict the scope of substrates, particularly those sensitive to basic conditions. Consequently, the development of catalysts that can operate under base-free and aerobic conditions has attracted significant attention, as these systems promise simpler procedures and broader applicability. In this context, Citta et al. reported a ruthenium catalyst capable of promoting the base-free transfer hydrogenation of ketones and aldehydes under aerobic conditions, while also demonstrating broad substrate scope (Scheme 13) [69].

Building on this, subsequent efforts have focused on expanding the range of metals and ligand frameworks capable of base-free transfer hydrogenation. Clarke and co-workers designed an iridium pincer complex that promotes ketone transfer hydrogenation without the need for an added base. This catalyst combines air-stability with high reactivity in 2-propanol, and its ability to operate efficiently under base-free conditions highlights the versatility of pincer-type ligands in broadening the applicability of practical transfer hydrogenation systems [70].

**Scheme 14:**
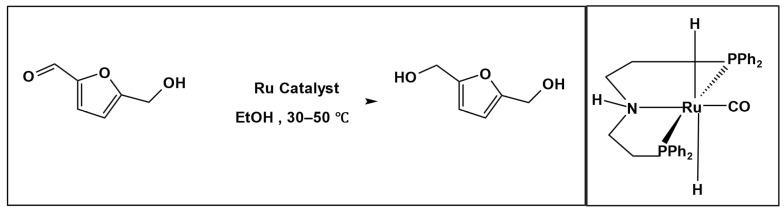
Catalytic transfer hydrogenation of Aldehyde using a ruthenium catalyst under base-free conditions.

Rosa Padilla et al. demonstrated a rapid and highly selective transfer hydrogenation method for furanic aldehydes using ethanol, isopropanol, or methanol as hydrogen donors. The Ru-MACHO-BH complex efficiently reduced these substrates under mild conditions without the need for additives, employing low catalyst loadings and showing excellent performance on a practical scale (Scheme 14) [71].

Heterogeneous catalysts have received growing attention in catalytic transfer hydrogenation (CTH), particularly as alternatives to noble-metal homogeneous systems that often suffer from limited recyclability and challenging separation. These solid catalysts—ranging from supported metals to metal oxides and hybrid materials—offer advantages such as facile recovery, high stability, and compatibility with greener reaction conditions.

**Scheme 15:**
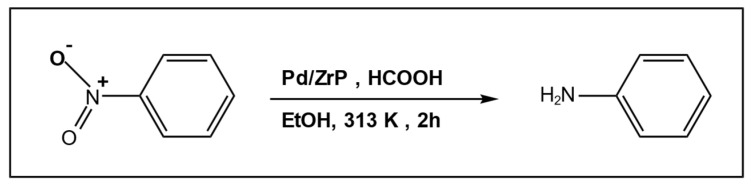
Catalytic transfer hydrogenation of nitroarines using a Pd/ZrP catalyst under base-free conditions.

In this context, Jaya Tuteja et al. [72] reported an efficient heterogeneous system based on Pd/ZrP for the base-free CTH of nitroarenes using formic acid as the hydrogen donor (Scheme 15). Their study demonstrates that Pd/ZrP can selectively reduce a variety of substituted nitroarenes to the corresponding anilines under mild and environmentally benign conditions. The catalyst not only operates without added base but also maintains high activity over multiple cycles, highlighting the potential of heterogeneous systems for sustainable CTH. The mechanistic investigation proposed by the authors—supported by CO_2_ detection and Hammett analysis—suggests a pathway involving FA decomposition on the catalyst surface, formation of a cationic intermediate, and strong electrostatic adsorption of nitroarenes, all of which contribute to the system’s high chemoselectivity.

Building on the industrial relevance of nitroarene reduction, it is important to note that anilines are key intermediates in pharmaceuticals, agrochemicals, dyes, polymers, and fine chemicals. Although heterogeneous noble-metal catalysts such as Ni and Pt are widely used for large-scale hydrogenations, they often show limited chemoselectivity, particularly toward substituted nitrobenzenes, which restricts their applicability. These challenges underscore the need for catalytic systems that combine selectivity, functional-group tolerance, and sustainability.

**Scheme 16:**
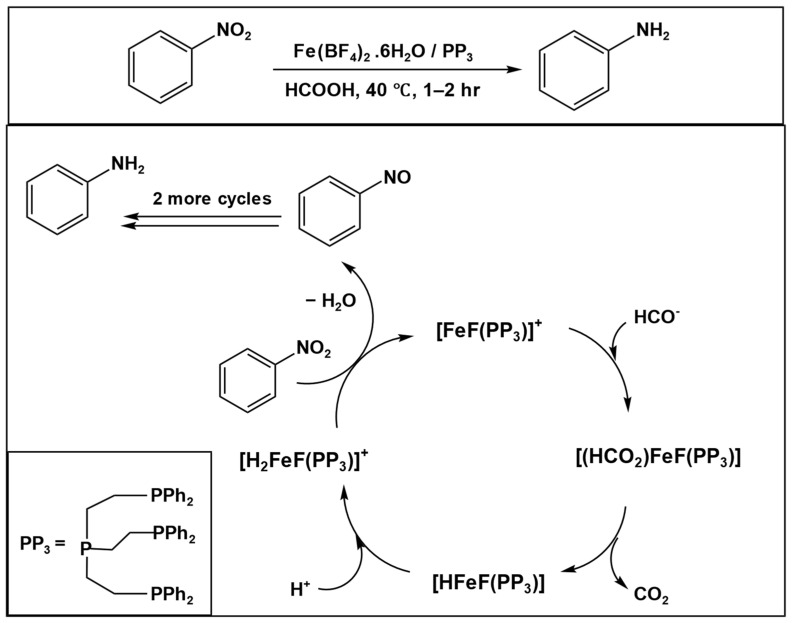
Proposed base-free transfer hydrogenation mechanism with a Fe catalyst [74].

In this context, homogeneous catalysts based on earth-abundant metals have provided valuable advances toward base-free transfer hydrogenation. A notable example is the system developed by Beller and co-workers, who reported an iron-catalyzed CTH of nitroarenes using formic acid as the sole hydrogen source, without requiring any basic additive [73]. As shown in (Scheme 16, the active cationic species [FeF(PP_3_)]^+^ directly coordinates formate to generate a neutral iron–formate complex, which undergoes β-hydride elimination to release CO_2_ and produce an iron dihydride intermediate. Crucially, this hydride species is generated without prior deprotonation by base, distinguishing the mechanism from classical formate–base systems. The resulting Fe–H species sequentially reduces nitrobenzene through nitroso and hydroxylamine intermediates—none of which accumulate, consistent with rapid, base-independent turnover.

Together, these findings show that carefully designed homogeneous catalysts can mediate efficient and chemoselective base-free transfer hydrogenation of nitroarenes, complementing earlier heterogeneous approaches and broadening the scope of mild CTH methodologies.

To emphasize the growing importance of base-free transfer hydrogenation, Table 8 below summarizes representative homogeneous and heterogeneous catalytic systems operating without external base, together with their hydrogen donors, substrate scope, mechanistic features, and key performance metrics.

In addition to the mechanistic schemes provided above, a comparative summary is presented in Table 9 to further highlight the distinctions between base-assisted and base-free, and pathway

## 3. Future Trends

In this review, we have highlighted the various roles of bases in catalytic transfer hydrogenation (CTH), including their functions as additives, co-hydrogen donors, and their absence in base-free systems. As the field continues to develop, several promising directions are emerging to enhance sustainability, expand substrate scope, and improve reaction efficiency. These trends underscore the growing interest in green chemistry, mechanistic innovation, and practical applicability in both academic and industrial settings.

### 3.1. Base-Free Catalytic Systems

Certain transition-metal catalysts have demonstrated efficient hydrogen transfer under neutral or nearly neutral conditions. Carefully engineered ligand environments can facilitate internal proton and hydride transfers, effectively replacing the need for external bases. Base-free systems are particularly advantageous for base-sensitive substrates, expanding the substrate scope while simplifying reaction protocols and reducing chemical waste. Future research will likely focus on understanding structure–activity relationships and designing ligands that maximize intrinsic catalytic activity.

### 3.2. Green Base Selection

For systems that still require a base, there is a clear shift toward recyclable and environmentally benign materials. Solid-supported, polymeric, and inorganic bases allow easy recovery and minimize metal contamination. Bio-derived amines and amino acid-based bases have also shown promising activity, suggesting potential for integration into fine chemical and pharmaceutical production. These developments align with broader trends in sustainable and circular chemistry.

### 3.3. Advanced Catalyst Design

Catalyst innovation remains a central driver of progress in CTH. Multifunctional ligands that internally replicate the role of an external base can enhance proton-coupled electron transfer and hydride migration. Pincer ligands, including PNP (phosphine-nitrogen- phosphine), NNN (tridentate nitrogen), and CNC (carbon-nitrogen-carbon) ligands, have demonstrated improved reactivity and selectivity through the incorporation of basic sites. Computational modeling, machine learning, and artificial intelligence could further accelerate the discovery and optimization of ligand scaffolds. These approaches are particularly valuable in asymmetric transfer hydrogenation (ATH), where data-driven design enables precise control over enantioselectivity and reaction efficiency.

### 3.4. Acid–Base Ratio Optimization

In formic acid/amine-based hydrogen donor systems, the acid–base ratio is a critical determinant of reaction efficiency. Real-time control of pH and buffer composition, potentially via microreactor technology and in situ spectroscopy, could help maintain optimal hydrogen release and prevent catalyst deactivation. This approach is especially relevant for aqueous-phase ATH and biomass-derived substrates, where acid–base equilibria change dynamically during the reaction.

### 3.5. Emerging Mechanistic and Technological Directions

CTH research can move beyond traditional heating methods toward alternative activation strategies, including electrocatalysis and photocatalysis, which combine hydrogen transfer with sustainable energy inputs. CTH can also be applied in sequential or multi-step reactions that integrate hydrogen transfer with other transformations, offering more efficient routes to complex molecules. Although large-scale applications in bulk chemical production remain limited, these developments emphasize the relevance of CTH in fine chemical and pharmaceutical synthesis.

## 4. Conclusions

Catalytic transfer hydrogenation (CTH) has established itself as a versatile and safer alternative to direct hydrogenation, particularly for the synthesis of fine chemicals and pharmaceutical intermediates. Bases play critical roles in these reactions: as additives that activate hydrogen donors, as co-hydrogen donors in combination with hydrogen-rich compounds, and, in some systems, catalysts can operate under base-free conditions.

As additives, bases promote reactivity by facilitating the formation of metal–alkoxide and metal–hydride intermediates, enabling efficient hydride transfer to substrates. Strong bases such as NaOH and iPrOK typically accelerate conversion, while weaker bases like K_2_CO_3_ can enhance selectivity under specific conditions. In co-hydrogen donor systems, bases form reactive anions (e.g., formate) that participate directly in hydrogen transfer, broadening mechanistic possibilities and supporting green chemistry objectives. Studies on asymmetric transfer hydrogenation (ATH) highlight the importance of base identity and acid–base ratios, where subtle changes in pH or base structure can significantly affect enantioselectivity and reaction rates.

Base-free catalytic platforms are emerging as a promising direction for addressing base-sensitive substrates. Transition metal complexes and supported catalysts based on ruthenium, iridium, palladium, and iron have demonstrated efficient reductions of aldehydes, ketones, imines, nitriles, and nitroarenes under mild, additive-free conditions, with high functional-group compatibility.

Future directions include the expansion of base-free systems, the development of greener or recyclable bases, and the design of multifunctional catalysts capable of internal proton–hydride management. Continued studies on base–ligand interactions, tunable metal–ligand cooperativity, stereoselectivity control, and sustainable reaction media are expected to further enhance both the efficiency and environmental compatibility of next-generation CTH processes.

Overall, CTH remains highly relevant for the synthesis of fine chemicals and pharmaceutical intermediates, where efficiency, selectivity, and environmental compatibility are particularly important.

## Figures and Tables

**Table 1:** Brief summary of the use of base in transfer hydrogenation.

Function of Base	How It Operates/Mechanistic Role	Reference
Activation of hydrogen donor (alcohol deprotonation/alkoxide formation)	The base deprotonates the alcohol, generating an alkoxide (or other activated donor) which is more susceptible to transfer hydrogenation or hydride formation. This tends to increase the reactivity of the hydrogen donor.	[17]
Catalyst activation (pre-activation, generation of active metal species)	Some metal catalyst precursors are in inactive or dormant forms; base helps convert them into the active form, e.g., by removing ligands like carbonyls, generating a metal hydride, or opening coordination sites.	[18]
Facilitation of hydride transfer or proton transfer steps	Base helps in the deprotonation (or proton shuttle) steps. In many “bifunctional” catalysts, there is a ligand with a proton-donor (–NH, –OH) site; base ensures these are in correct tautomeric or protonation state; may assist in regenerating ligand protonation states, etc.	[19]
Adjusting thermodynamics and kinetics	By affecting the acidity/basicity of the medium, the base influences equilibrium positions (e.g., of alcohol dehydrogenation), rate constants of dehydrogenation/hydride transfer, of proton transfer etc. Also, base can stabilize or destabilize intermediates/transition states. Solvation effects etc. matter.	[20]
Influence on selectivity, especially enantio- or diastereo-selectivity	The base may interact with or coordinate to catalyst or substrate, influencing binding orientation or TS geometry; it may compete with substrate for binding or affect supramolecular structure of TS.	[21]

**Table 2:** Effect of Different Bases on the Transfer Hydrogenation of acetophenone using Ru catalyst [28].

Base	Catalyst	Time	Yield (%)	Interpretation
iPrOK	4	10 min	94	Efficient activation of catalyst 4 due to strong, less hindered alkoxide.
iPrOK	5	10 min	70	Catalyst 5 is sterically hindered → slower alkoxide formation.
iPrOK	4	1 min	79	Rapid activation, but early-time yield still kinetically limited.
tBuOK	4	1 min	68	Bulky base slows formation of metal–alkoxide.
tBuONa	4	1 min	76	Sodium cation improves ion pairing compared to potassium.
KOH	4	1 min	96	Strong, non-hindered base → fast alcohol deprotonation.
NaOH	4	1 min	96	Same as KOH; strongest performers at short reaction time.
No base	4 and 5	10 min	0	No reaction without alkoxide formation.

**Table 3:** Effect of Different Bases on the Transfer Hydrogenation of acetophenone using Co catalyst.

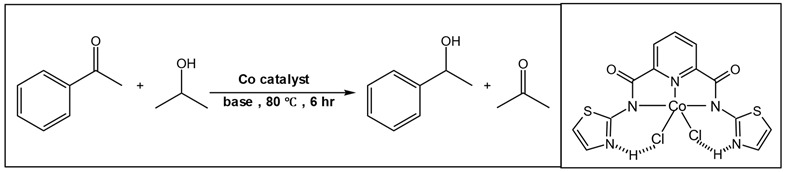		
Base	% Yield	PKa
KOH	>99	>14
NEt_3_	15	10.75
KOtBu	30	18
NaOH	40	>15

**Table 4:** Effect of Different Bases on the Transfer Hydrogenation of acetophenone using Mn catalyst [39].

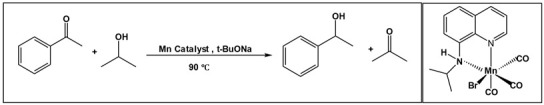			
Base	Counter-Ion	Conversion (%)	Mechanistic Insights
t-BuONa	Na^+^	99	Highest activity; strong, soluble alkoxide enabling rapid alkoxide–hydride cycling.
t-BuOK	K^+^	97	Slightly lower than Na^+^ analogue, supporting Na^+^ > K^+^ trend in promoting active Mn–hydride formation.
NaHMDS	Na^+^	91	Strong non-nucleophilic base; efficient at generating Mn–alkoxide intermediates.
KHMDS	K^+^	86	Lower than NaHMDS; consistent counter-ion effect.
NaOH	Na^+^	94	Strong inorganic base; effective hydride-forming pathway.
KOH	K^+^	91	Slightly lower than NaOH; further supports Na^+^ > K^+^ trend.
Ca(OH)_2_	Ca^2+^	4	Poor solubility/basicity under these conditions → minimal Mn activation.
Ba(OH)_2_	Ba^2+^	14	Similar limitations; inadequate to generate active Mn–hydride species.

**Table 5:** Summary of catalytic systems operating under base-assisted transfer hydrogenation conditions.

Catalyst	Substrate Type	H-Donor	Role of Base/Mechanistic Insight	Yield (%)	Reference
Core–shell Ag@Ni magnetic nanocatalyst	Aromatic nitro and ketone compounds	Isopropanol	KOH generates isopropoxide, enhancing hydride availability at the metal surface. Reduction proceeds via nitro → nitroso → hydroxylamine → aniline pathway through successive surface-mediated hydride transfers.	Up to 90%	[40]
Ruthenium complexes	Benzaldehyde	Glycerol	Strong inorganic base required to deprotonate glycerol and enable Ru–hydride formation. Organic amine bases are ineffective, highlighting the need for high basicity to activate glycerol as an H-donor.	Up to 48%	[41]
RuCl_2_(PPh_3_)_3_	Ketones	Secondary alcohols	K_2_CO_3_ required only during the initial step to deprotonate the alcohol and generate Ru–dihydride species (RuH_2_(PPh_3_)_4_). Once formed, catalysis proceeds without further base assistance.	>90%	[42]
Nickel aluminosilicate	Ketones	Isopropanol	KOH acts as a basic promoter, facilitating alcohol dehydrogenation and formation of hydride species at the catalyst surface, thereby enhancing CTH activity.	Up to 98%	[43]
Pd catalyst	Aromatic alkynes	Formic acid	Triethylamine forms formate (HCOO^−^), enabling Pd-assisted hydride transfer from HCOO^−^ and promoting selective carbonyl reduction.	99%	[44]
Ru(II) carbonyl complexes with 1,8-naphthyridine ligands	Ketones	Isopropanol	NaOH and t-BuOK promote activation of the coordinated isopropanoxy ligand to form Ru–hydride species. NaOH is more effective; steric hindrance from t-BuOK reduces hydride formation and catalytic turnover.	66%	[45]
Iridium complex	Ketones	Isopropanol	KOH removes the halide ligand, generating a vacant coordination site and promoting formation of Ir–hydride and Ir–dihydride species. High basicity is essential; lower base loading suppresses hydride formation and decreases conversion.	Up to 93%	[46]
Ni-based homogeneous systems	Ketones	Isopropanol	KOH generates the corresponding Ni–alkoxide, which undergoes β-hydride migration to produce a Ni–hydride intermediate proposed as the active reducing species.	48–97% depending on ligand	[47]

**Table 6:** Effect of Base Type on the Rate and Selectivity of ATH of (R)-1,4-Dimethyl-3,4-Dihydroisoquinoline in Water [21].

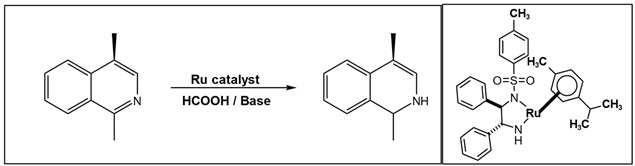			
Amine Type	Amine Name	Diastereoselectivity (%)	Pka
Tertiary	TEA	55.5	10.7
Tertiary	DIPEA	46.1	11
Tertiary	DABCO	63.8	8.8
Secondary	Morpholine	65.6	8.3
Secondary	Piperidine	65.1	11.2
Secondary	Pyrrolidine	71.5	11.3
Aromatic	Pyrrole	65.5	17
Aromatic	Imidazole	0	4.6
Aromatic	Pyridine	69.3	5.2

**Table 7:** Effect of Different Bases on the Transfer Hydrogenation of Benzonitrile Using Pd/C.

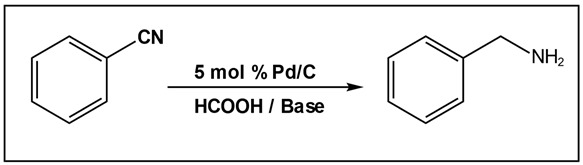			
Base	% Yield	Interpretation	pK_a_H (Approx.)
Triethylamine	98	Ideal balance of basicity + low steric hindrance	10.7
N-butylamine	98	Strong enough and unhindered → efficient activation	11.0
N-methylpyrrolidine	79	Slight steric constraint, moderately strong base	10.3
Hünig’s base (DIPEA)	77	Very bulky, less efficient despite moderate pK_a_	11.0
DBU	74	Very strong base; overbasicity may hinder Pd interactions	13.5

**Table 8:** Summary of catalytic systems operating under base-free transfer hydrogenation conditions.

Catalyst	Substrate	H-Donor	Mechanistic Insights	% Yield	Reference
IrH(CO)(PPh3)3	Aromatic Ketones	2 propanol	Chiral PNNP ligand enables internal metal–ligand bifunctional activation, allowing hydride formation and transfer without any added base.	>90%	[74]
Ru–p-cymene–pyrazolyl complex	benzophenone		Partial NN-ligand decoordination enables isopropanol activation → Ru–H monohydride formation → internal pyrazole base facilitates hydride generation under base-free conditions	>99%	[75]
MOF-derived Co@NC	Nitriles	Isopropanol	N-doped sites in the Co@NC catalyst act as intrinsic basic centers, promoting isopropanol deprotonation and proton transfer to the nitrile. These sites regulate the steady-state concentration of the imine intermediate, enabling a switchable pathway toward either primary amines or imines	>99	[76]
MMIO (micro–mesoporous Fe oxide)	Nitroarenes	Formic acid	Surface Fe cations form Fe–formate species with PP_3_, which decompose to generate the active Fe–hydride responsible for nitro reduction. The reaction proceeds via nitroso and hydroxylamine (or a hydrazo) intermediate, and hydride formation occurs without added base due to intrinsic basic sites on the Fe surface.	>90	[77]
Ru–salicylaldimine complexes	Aromatic ketones	Isopropanol	Base-free inner-sphere oxidative-addition mechanism: Ru undergoes oxidative addition with iPrOH to form Ru(IV)–alkoxide, then reductive elimination and β-H elimination generate the Ru–hydride. Ketone inserts into the Ru–H bond, and a second oxidative addition regenerates the active species.	90	[78]
Pd/Al_2_O_3_	benzonitriles	Formic acid	FA dissociates on Pd surface to give H^+^(ads) and CO_2_H^−^(ads). Benzonitrile undergoes stepwise cationic reduction (supported by negative Hammett ρ): R–C≡N → R–C^+^=NH → R–CH=NH → R–CH^+^–NH_2_ → R–CH_2_–NH_2_, with CO_2_ released during formate-assisted hydride steps.	>96%	[79]

**Table 9:** Key mechanistic differences between base-assisted and base-free transfer hydrogenation pathways.

Feature	Base-Assisted CTH	Base-Free CTH
Hydride Formation	Generated via external base: M–OR formation → β-H elimination → M–H	Generated intrinsically via ligand cooperation, metal–ligand bifunctionality, bifunctional surfaces
Source of Basicity	External base required (KOH, t-BuOK, Cs_2_CO_3_, TEA)	Internal basicity from ligand N-sites, pincer arms, or metal–support interfaces
First Key Step	Deprotonation of alcohol or FA → metal alkoxide/formate	Ligand hemilability, bifunctional activation, or surface-assisted H-donor activation
Rate-Determining Step	β-H elimination from M–OR (classical)	Proton/hydride transfer (ligand- or surface-mediated)
Substrate Binding Mode	Classical coordination to metal hydride	Often outer-sphere, ligand-assisted, or metal–support cooperative binding
Catalyst Requirements	Catalyst must tolerate strong base; halide loss often needed for activation	Catalyst must include intrinsic bifunctional or redox-active motifs
Advantages	Rapid hydride formation → high TOF and ee%	No external base → greener, compatible with acid-sensitive groups
Limitations	Not compatible with base-sensitive substrates; requires stoichiometric base	Often requires sophisticated ligand or support design

## Data Availability

No new data were created or analyzed in this study.
